# A two-step screening platform based on *trans*-aconitic acid assimilation unlocks novel bacterial resources for *trans*-aconitic acid production

**DOI:** 10.1128/aem.01831-25

**Published:** 2026-04-13

**Authors:** Cao Zheng, Jingyi Hua, Chengru Yang, Bowen Sun, Jingru Wu, Anming Li, Yujun Dai, Cuiying Du

**Affiliations:** 1 Hubei Province Research Center of Engineering Technology for Utilization of Botanical Functional Ingredients & Hubei Key Laboratory of Resource Utilization and Quality Control of Characteristic Crops, College of Life Science and Technology, Hubei Engineering University 162803, Xiaogan, Hubei, China; Chalmers tekniska hogskola AB, Gothenburg, Sweden

**Keywords:** *trans*-aconitic acid, *trans*-aconitic acid production, carbon source assimilation, high-throughput screening platform, aconitate isomerase, directed screening, microbial resource

## Abstract

*trans*-Aconitic acid (TAA) possesses significant advantages in bioactivity, biosafety, and chemical modifiability, leading to its wide application in biopesticides, pharmaceuticals, and biobased synthesis. However, the scarcity of microbial resources for TAA production limits its large-scale commercial production, making it difficult to replace currently costly and low-yield chemical synthesis and plant extraction methods. In bacteria, aconitate isomerases (AIs) catalyze the biosynthesis of TAA. AIs are bifunctional enzymes that can also facilitate the reverse reaction, using TAA as the sole carbon source for bacterial growth. This provides a readily observable marker for the selection of TAA-producing bacteria. Using this dual functionality, we developed a novel two-step screening strategy comprising: (i) high-throughput screening of TAA-assimilating strains using a minimal medium with TAA as the sole carbon source and (ii) secondary screening of these assimilating strains to detect their ability to produce TAA in citrate medium. A total of 219 TAA-assimilating bacteria were isolated from a variety of environmental samples (*n* = 135). Subsequent LC-Q-TOF-MS analysis identified 19 strains (8.68%) capable of producing TAA at concentrations ranging from 34.5 to 621.9 μM. Taxonomic analysis revealed two new orders, five new families, and seven new genera of TAA-producing bacteria. This study established an efficient AI-directed strategy for discovering novel TAA-producing bacteria and significantly expanded taxonomic diversity, providing a sustainable resource for microbial technology development and strain innovation, thereby promoting the industrial biomanufacturing of TAA and its applications in multiple fields.

**IMPORTANCE:**

This study proposes a novel method for constructing a high-throughput screening platform using aconitate isomerase, which catalyzes both the biosynthesis and assimilation of *trans*-aconitic acid (TAA), to target the discovery of microbial TAA-producing strains. Using TAA assimilation as a phenotypic marker for rapid screening, this strategy overcomes the key bottleneck of scarce microbial resources. The results show that this method effectively identified seven previously unknown genera of TAA-producing bacteria, greatly expanding the phylogenetic diversity and revealing the widespread presence of TAA metabolism across different taxa. Significant yield variation among different isolates underscores their potential as chassis for strain improvement. These resources provide a sustainable driving force for constructing efficient microbial cell factories, facilitating industrial bioproduction of TAA to meet the growing demands of agricultural biocontrol and advanced biomanufacturing.

## INTRODUCTION

**Fig 1:**
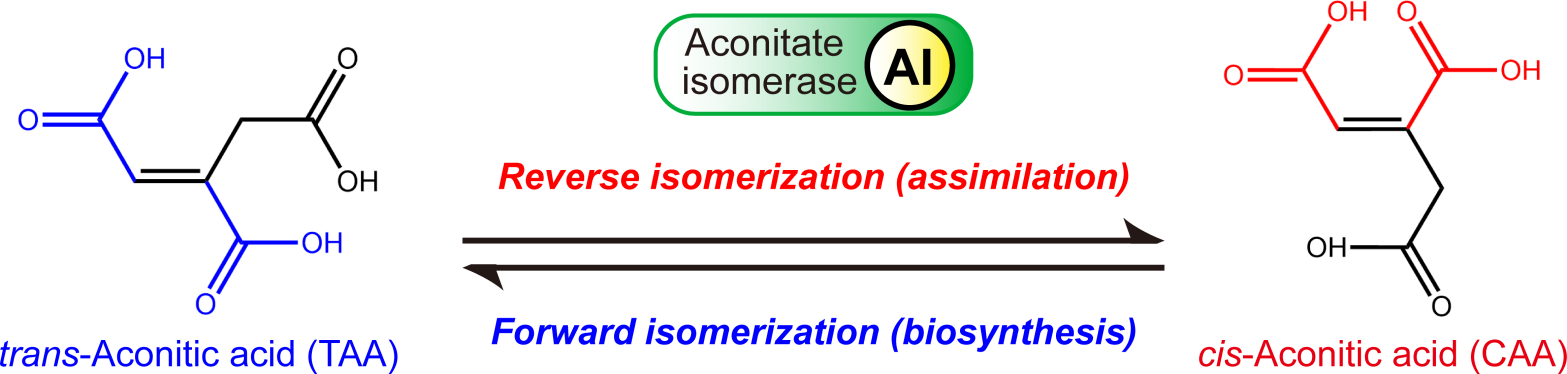
Enzymatic mechanism of AI-mediated TAA biosynthesis and assimilation in bacteria.

*trans*-Aconitic acid (TAA) is an unsaturated tricarboxylic acid, a geometric isomer of *cis*-aconitic acid (CAA), a key intermediate in the tricarboxylic acid (TCA) cycle (Fig. 1) (1). TAA is naturally found in plants (2–4), such as maize, sugarcane, and sorghum (5–7), typically constituting a large portion of the plant’s dry weight, for example, 3.5% in barley (*Hordeum leporinum*), 4.2% in reed grass (*Phalaris tuberosa*), and up to 12.2% in western larkspur (*Delphinium hesperium*) (2, 8). Therefore, it is biologically safe for mammals (9) and has been approved as a food additive by the World Health Organization (WHO) and the United States Food and Drug Administration (U.S. FDA). Moreover, due to its conformational properties, such as unsaturation and abundant carboxyl groups, TAA is widely used in chemical engineering as a polymer monomer, antioxidant, plasticizer, and lubricant additive (6, 10) and has been rated as one of the 30 most promising building blocks. However, because it has not yet been commercially produced, it was ultimately excluded from the final list of 12 most promising biobased chemicals (11).

As a biomolecule, TAA possesses specific biological activities and can be obtained by biosynthesis. As early as 1975, it was discovered that TAA has an antifeedant effect against brown planthoppers (one of the most destructive pests in rice) (12, 13). Since 1989, it has been found that TAA has a potent inhibitory activity on the growth and morphological transformation of *Leishmania* parasites, as well as a significant therapeutic efficacy on leishmaniasis in mammals (9, 14). In 2010, the rich content of TAA molecules and its antiedema effect in *Echinodorus grandiflorus*, a traditional Brazilian herb used to treat inflammatory diseases, was confirmed (15, 16). Recently, our group discovered the excellent nematicidal activity of TAA in the study of *Bacillus thuringiensis* (17), a well-known insecticidal bacterium that has been commercialized in the field of agricultural biocontrol worldwide (18, 19). However, the high costs and low yields of TAA severely limit its practical applications (20). TAA is primarily produced through chemical synthesis or plant extraction (21, 22), methods generally suffer from complex processes, low efficiency, low conversion rates, and unavoidable by-product generation. Therefore, biological production of TAA holds promise for overcoming these obstacles and promoting its application.

However, the first TAA biosynthetic pathway and its associated genes, namely, the *tbrAB* (TAA biosynthesis-related genes *A* and *B*) operon, were not elucidated until 2017 in *B. thuringiensis* (17). This breakthrough rapidly triggered the application of TAA in the biocontrol of plant parasitic nematodes, a major challenge facing global agriculture (23, 24). By expressing the TAA synthase TbrA, researchers successfully constructed a high-yield TAA-producing *B. thuringiensis* strain, HAN055, and registered it as the world’s first biopesticide targeting root-knot nematodes, which has been commercially applied in China since 2022. The application significantly reduces chemical pesticide while also bringing considerable economic benefits. Furthermore, by heterologously introducing the bacterial *tbrA* gene and optimizing the industrial fermentation process, an unprecedented yield of 60 g·L⁻¹ TAA was achieved in *Aspergillus terreus* (21). This evidence suggests that elucidating the genetic elements and microbial resources associated with TAA biosynthesis plays a pivotal role in unlocking the potential and key applications of TAA.

In bacteria, the biosynthesis of TAA is mediated by aconitate isomerase (AI, EC 5.3.3.7) (25), such as TbrA in *B. thuringiensis* (17), which catalyzes the isomerization of intracellular CAA to TAA (i.e., the forward reaction). TAA is then exported extracellularly and accumulated via specific transporters, such as TbrB (Fig. S1) (17). In practice, AI catalyzes the interconversion between CAA and TAA with a thermodynamic preference for TAA formation (26, 27). Interestingly, when TAA importers (such as TarB, as identified in *B. velezensis*) (1) are present on the cell membrane, AI predominantly catalyzes the reverse isomerization, converting environmental TAA substance back into CAA and supporting the growth of many bacteria, such as *B. velezensis* (1) and *Pseudomonas putida* (28) on minimal aconitic acid (ACO) medium with TAA as the sole carbon source. This metabolic diversity not only enhances the bacteria’s adaptability to resource-limited environments (1) but more significantly, it provides a readily observable “TAA-dependent growth” phenotype and a powerful targeted screening strategy for identifying potential AI-positive strains.

However, to date, only two wild-type bacterial isolates have been reported to produce TAA: *B. thuringiensis* CT-43 (17) and *Pseudomonas* sp. WU-0701 (29). Given that plant-derived TAA biosynthetic genes have not yet been identified, there is an urgent need to expand the taxonomic diversity of bacterial TAA-producing strains and their associated AI enzymes.

**Fig 2:**
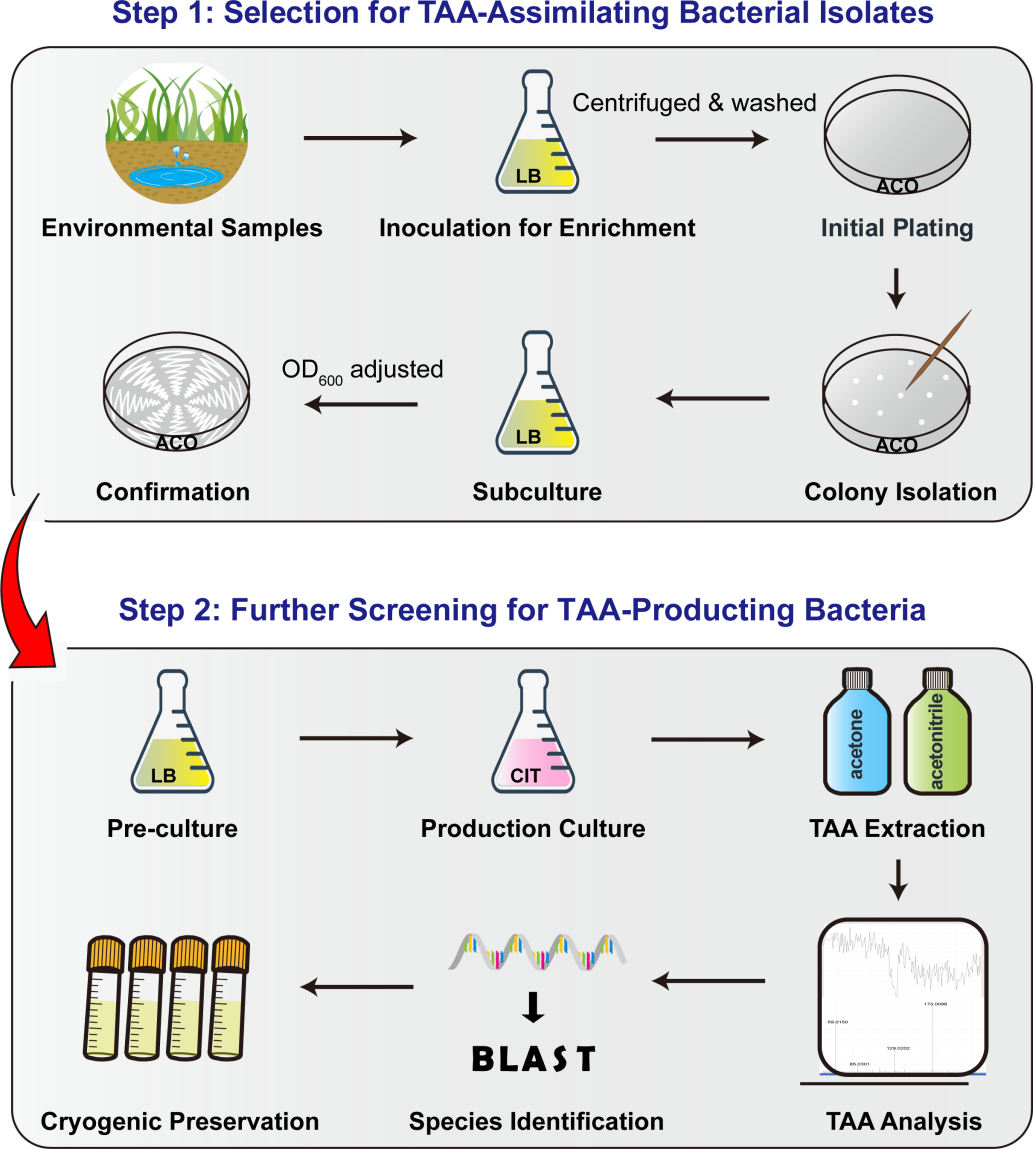
Schematic diagram of a two-step screening strategy for TAA-producing bacteria. Step 1: environmental samples (solid or liquid) are inoculated into lysogeny broth (LB) to enrich microorganisms, which were centrifuged, washed with ultrapure water to remove residual nutrients, and then spread onto minimal ACO plate. Distinct colonies were picked and inoculated into LB. The cultures are centrifuged and washed as described above, and the OD_600_ value is normalized to 0.01. This standardized suspension is then restreaked onto fresh minimal ACO plates to reconfirm its TAA assimilation capability. Step 2: the TAA-assimilating strains isolated in step 1 are precultured in LB and transferred into citric acid (CIT) liquid medium at a ratio of 1:100. After 24 h, the supernatant is extracted with acetone and acetonitrile. The pellets are dissolved in mobile phase, and the presence and concentration of TAA are analyzed. The final target strains are preserved in a 25% (vol·vol⁻¹) glycerol solution at −80°C.

Here, we report a highly efficient two-step screening method for identifying TAA-producing bacteria. The strategy first performs high-throughput screening of TAA-assimilating isolates, followed by targeted chromatographic analysis to confirm TAA production (Fig. 2). Using this approach, we successfully identified two new orders (*Enterobacterales* and *Moraxellales*), five new families (*Erwiniaceae*, *Yersiniaceae*, *Enterobacteriaceae*, *Morganellaceae*, and *Moraxellaceae*), and seven new genera (*Pantoea*, *Serratia*, *Erwinia*, *Enterobacter*, *Citrobacter*, *Providencia*, and *Acinetobacter*) of TAA-producing bacteria from various environmental samples. Our study establishes a robust and efficient screening strategy, significantly expanding the diversity of known TAA-producing bacterial resources.

## RESULTS

### Diverse environmental bacteria exhibit gradient phenotypes in TAA assimilation

**Fig 3:**
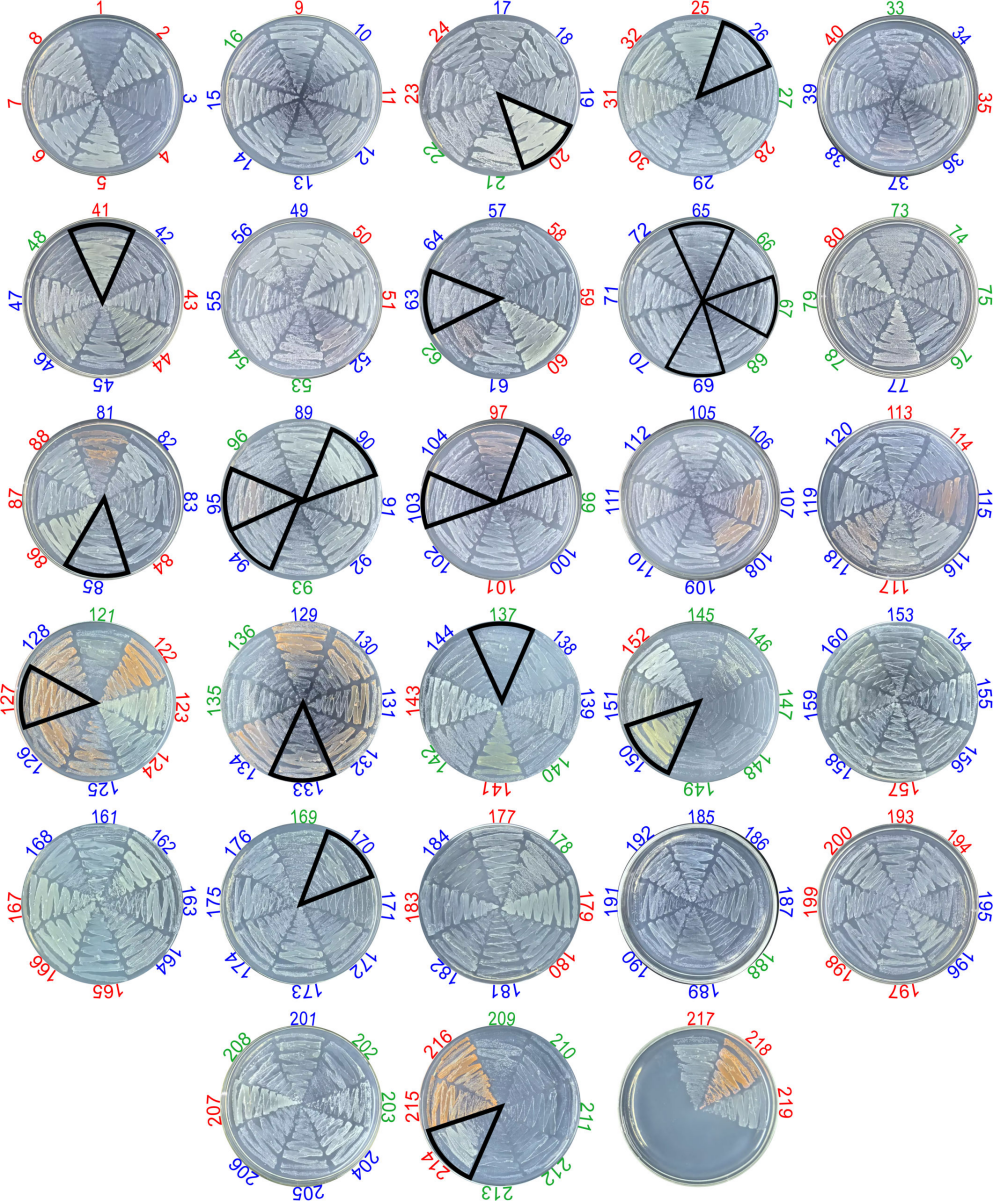
Growth of 219 TAA-assimilating bacterial isolates on minimal ACO plates. Different colors are used to encode different TAA assimilation efficiencies. Red indicates high efficiency with vigorous lawn growth; blue represents moderate efficiency with partial lawn formation; green denotes low efficiency with scattered colonies. The 19 TAA-producing bacterial isolates are marked with triangles with black borders.

Using minimal ACO medium, 219 bacterial strains capable of using TAA as the sole carbon source were isolated from 135 diverse environmental samples (Table S1). These isolates exhibited differential growth efficiencies, indicating differences in their TAA assimilation efficiency (Fig. 3). Based on this growth heterogeneity, we divided the isolates into three qualitative groups: “strong assimilation” (65/219, 29.68%), “moderate assimilation” (111/219, 50.69%), and “weak assimilation” (43/219, 19.63%) (Fig. 3).

### Nineteen TAA assimilation isolates are confirmed to be extracellular TAA-producing bacteria

**Fig 4:**
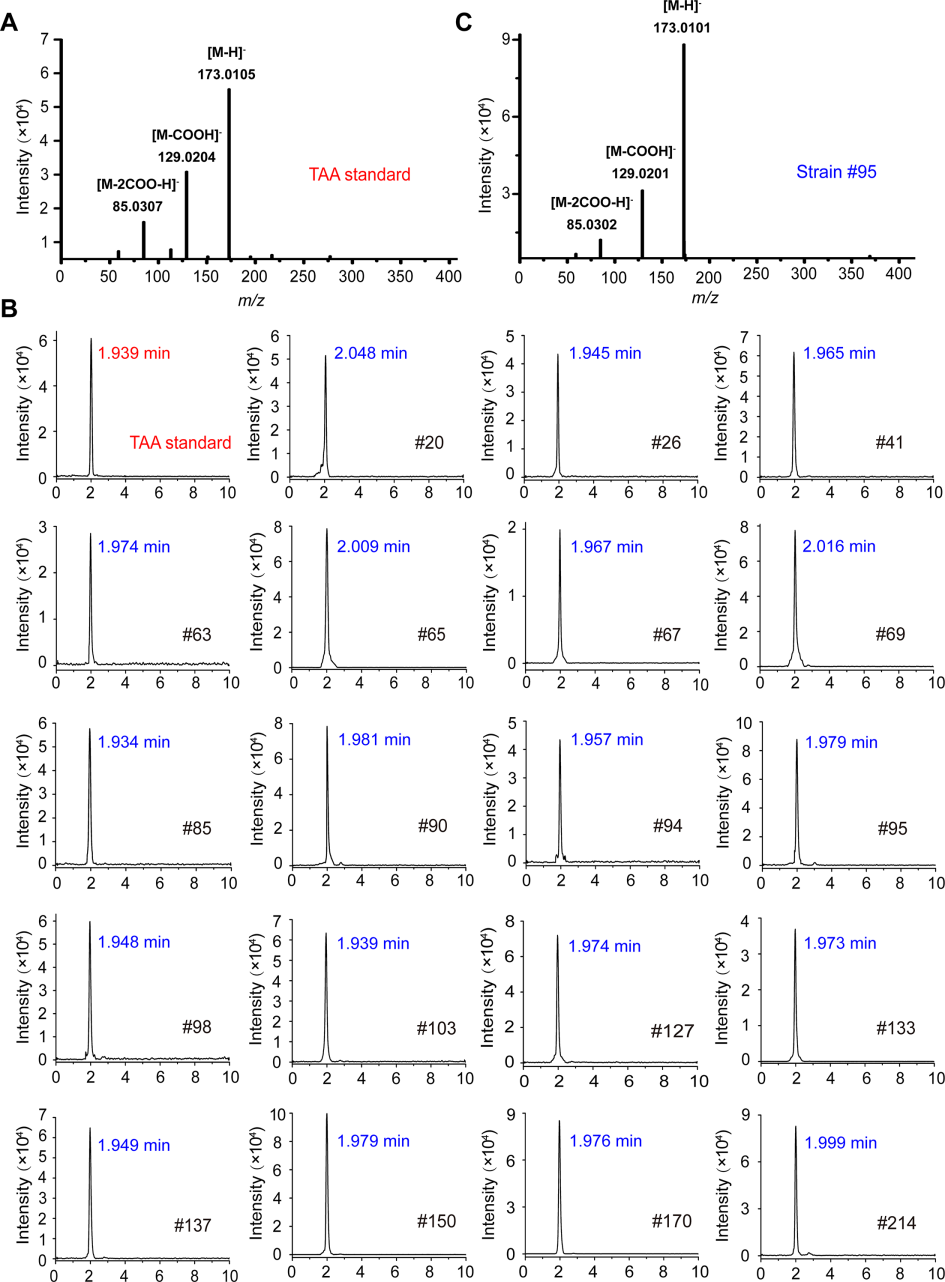
LC-Q-TOF-MS validation of TAA production in 19 positive isolates. (**A**) Mass spectrum of TAA standard showing the [M-H]⁻ ion at *m*/*z* 173.0105 and its sequential decarboxylation products [M-COOH]⁻ at *m*/*z* 129.0204 and [M-2COO-H]⁻ at *m*/*z* 85.0307. (**B**) Extraction ion chromatograms of TAA extracts from 19 positive strains at *m*/*z* 173.0105. (**C**) Representative mass spectrum of TAA extract from strain #95.

To determine TAA production capacity, we analyzed the culture supernatant extracts of 219 TAA-assimilating isolates cultured in citric acid (CIT) liquid medium using LC-Q-TOF-MS. The TAA standard has three characteristic ions: [M-H]⁻ at *m*/*z* 173.0105, [M-COOH]⁻ fragment at *m*/*z* 129.0204, and [M-2COO-H]⁻ fragment at *m*/*z* 85.0307 (Fig. 4A). Ions at *m*/*z* 173.0105 in the total ion chromatograms of 219 samples were screened, and the results showed that there were obvious TAA product peaks in 19 isolates, and the retention time of each peak was consistent with the retention time of TAA standard (Fig. 4B). The presence of both [M-COOH]⁻ (129.0201) and [M-2COO-H]⁻ (85.0302) ions in the mass spectra, consistent with the standard fragment spectrum of TAA, further confirmed this finding (Fig. 4C; Fig. S2). These results demonstrate the successful identification of 19 TAA-producing strains (19/219, 8.68%).

**Fig 5:**
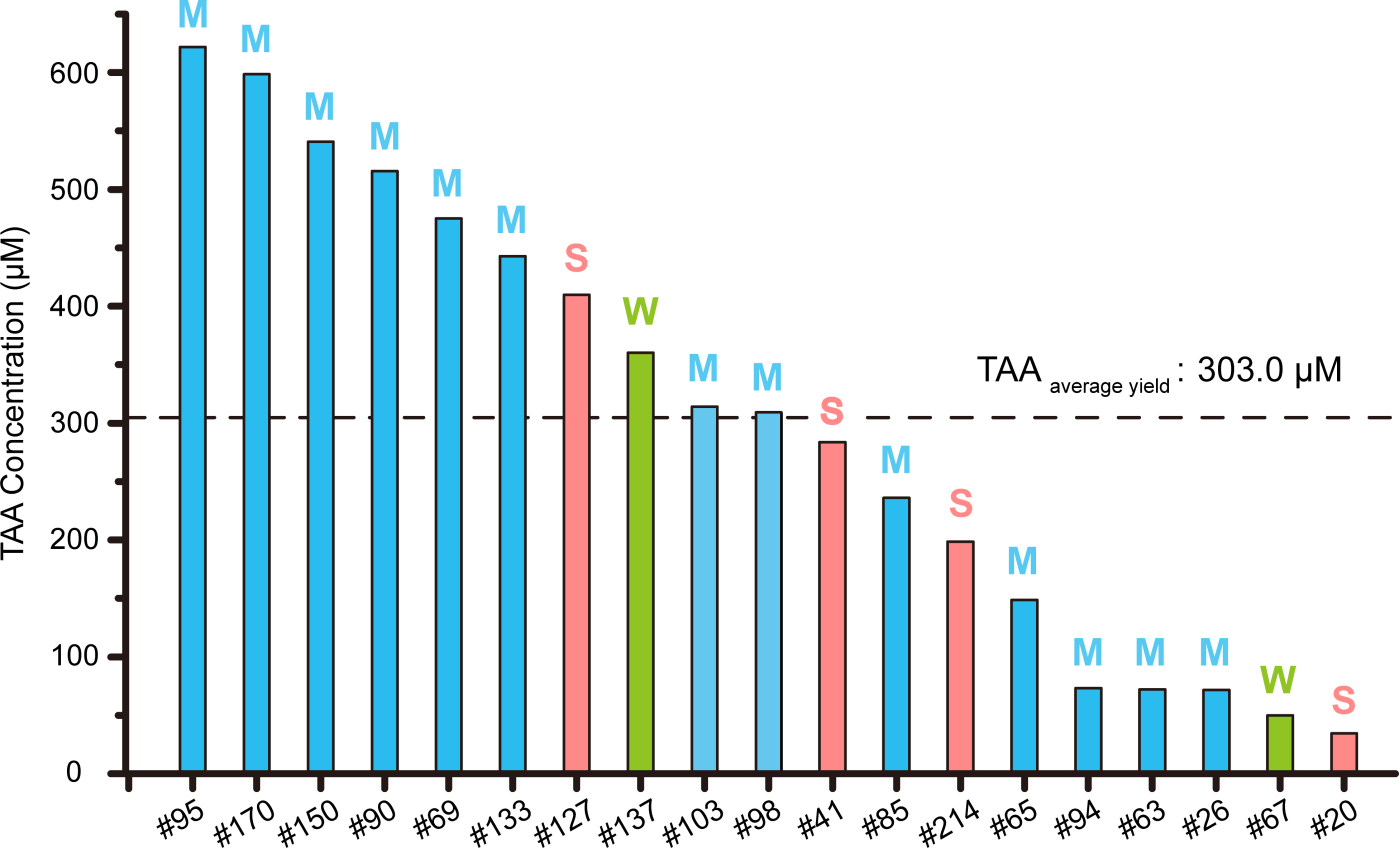
TAA production yields of 19 positive isolates. The strains are arranged in descending order of TAA yield, and their TAA assimilation efficiency on ACO plates is represented by color: red represents strong assimialtion (“S”); blue represents moderate assimilation (“M”); and green represents weak assimilation (“W”).

Analysis of the isolation sources of these 19 TAA-producing bacteria revealed a notable correlation with the sampling habitats (Fig. 5; Table S1). Most strains were isolated from rhizosphere soil (11/19, 57.9%; 10 positive samples/96 total samples, detection rate 10.42%) and plant tissues (5/19, 26.3%; 4 positive samples/18 total samples, detection rate 22.22%). Strikingly, the five highest-producing isolates all originated from plant-associated environments, particularly gramineous plants such as maize and grasses, which are known natural accumulators of TAA. This group included the top-producing strain #95 (*Pseudomonas paraglycinae*, “maize rhizosphere soil”), #170 (*Pantoea dispersa*, “straw bale,” rank second), #150 (*Serratia bockelmannii*, “rhizosphere soil,” rank third), #90 (*Pantoea anthophila*, “Bermuda grass,” rank fourth), and #69 (*Pseudomonas sp002113045*, ‘maize leaf’, rank fifth). In contrast, samples from other habitats showed far fewer TAA-producing strains and lower yields, with detection rates of 33.33% (1/3) in animal feces, 8.33% (1/12) in water, and 0% (0/5) in sludge.

### TAA production capacity is independent of assimilation efficiency

Quantitative analysis of TAA content in CIT medium revealed significant differences in yield levels among the 19 isolates, ranging from 34.5 to 621.9 μM (Fig. 5; Fig. S3). Pearson correlation analysis of these strains showed no significant correlation between TAA assimilation performance and yield (*P* = 0.962) (Fig. 3 and 5). For example, isolate #95 exhibited moderate TAA assimilation capacity but the highest TAA yield (621.9 μM), which was 18 times higher than isolate #20 (34.5 μM), which had strong assimilation capacity but the lowest TAA yield (Fig. 3 and 5). Similarly, isolate #137, with weak assimilation capacity (Fig. 3), also achieved a TAA yield of 360.2 μM, significantly higher than the average yield of 303.0 μM (Fig. 5). These results indicate that TAA assimilation capacity does not directly determine TAA yield.

### Taxonomic analysis expands the known diversity of TAA-producing bacteria to new orders, families, and genera

All 19 TAA-producing isolates were successfully identified. Taxonomic analysis showed that they all belong to the class *Gammaproteobacteria* and the phylum *Pseudomonadota* (Table S2). However, apart from four isolates*—P. paraglycinae* (strains #95 and #137), *Pseudomonas sp002113045* (strain #69), and one potential novel *Pseudomonas* species (strain #85)—the remaining 15 strains were distributed in two new TAA-producing orders: *Enterobacterales* and *Moraxellales*; five new families: *Erwiniaceae*, *Yersiniaceae*, *Enterobacteriaceae*, *Morganellaceae*, and *Moraxellaceae*; and seven new genera: *Pantoea* (*n* = 3), *Serratia* (*n* = 6), *Erwinia* (*n* = 1), *Enterobacter* (*n* = 1), *Citrobacter* (*n* = 1), *Providencia* (*n* = 2), and *Acinetobacter* (*n* = 1) (Table S2).

**Fig 6:**
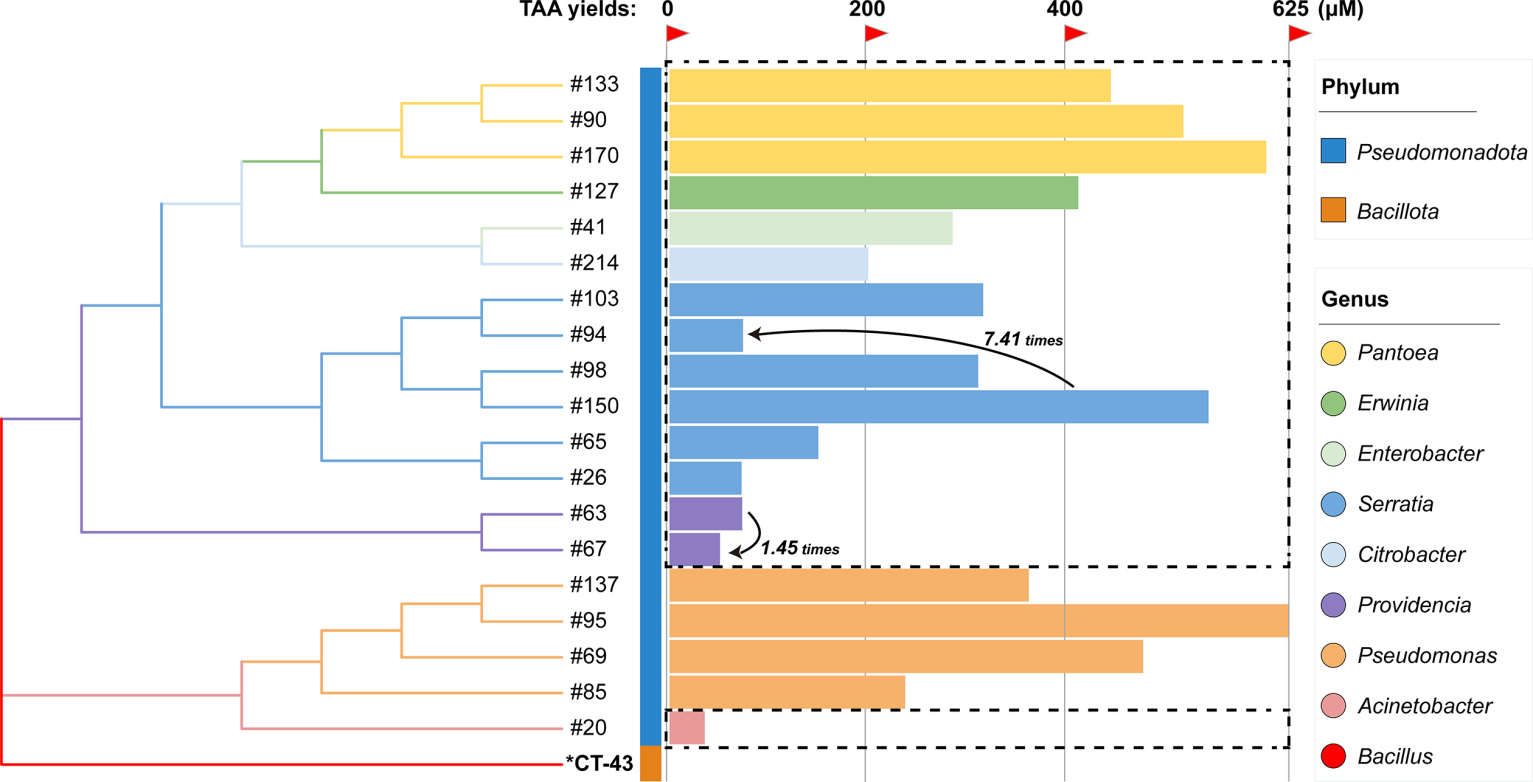
Phylogenetic analysis and TAA yield assessment of TAA-producing bacterial strains. TAA production levels are represented by horizontally adjacent bar plots. Dashed rectangles highlight the 15 isolates distributed across seven new TAA-producing genera. Intraspecific variations in TAA yield are quantified as the ratio of maximum to minimum yield among strains within the same species.

Of the only two reported TAA-producing bacteria*—B. thuringiensis* CT-43 (17) and *Pseudomonas* sp. WU-0701 (29)—only the genome sequences of the former have been published (30). Phylogenetic analysis of these newly isolated TAA-producing bacteria revealed that the dominant producers, such as the *Pantoea* and *Serratia* groups, are phylogenetically distant from *B. thuringiensis* CT-43 (Fig. 6). Furthermore, significant intraspecific differences in TAA yield were observed, ranging from 1.45- to 7.41-fold. A typical example is *S. bockelmannii* strains #150 and #94, with #150 exhibiting a 7.41-fold higher TAA production than #94 (Fig. 6). This finding indicates inherent differences in TAA biosynthetic efficiency among intraspecific strains, highlighting the need for further strain-level screening to identify high-yield producing strains.

## DISCUSSION

To our knowledge, this study is the first to conduct large-scale targeted screening of TAA-producing bacteria. We established a simple and efficient screening method using TAA assimilation phenotype as a key AI indicator. This approach identified TAA-producing bacteria comprising two new orders, five new families, and seven new genera from different ecological niches and sample types, validating the robustness and practicality of the screening platform. Based on the observed detection pattern, our data suggest that future efforts should prioritize sampling of plant-associated environments, particularly the rhizosphere and plant tissues of natural TAA-accumulating plants, such as *Poaceae* species, to efficiently identify high TAA-producing microorganisms.

**Fig 7:**
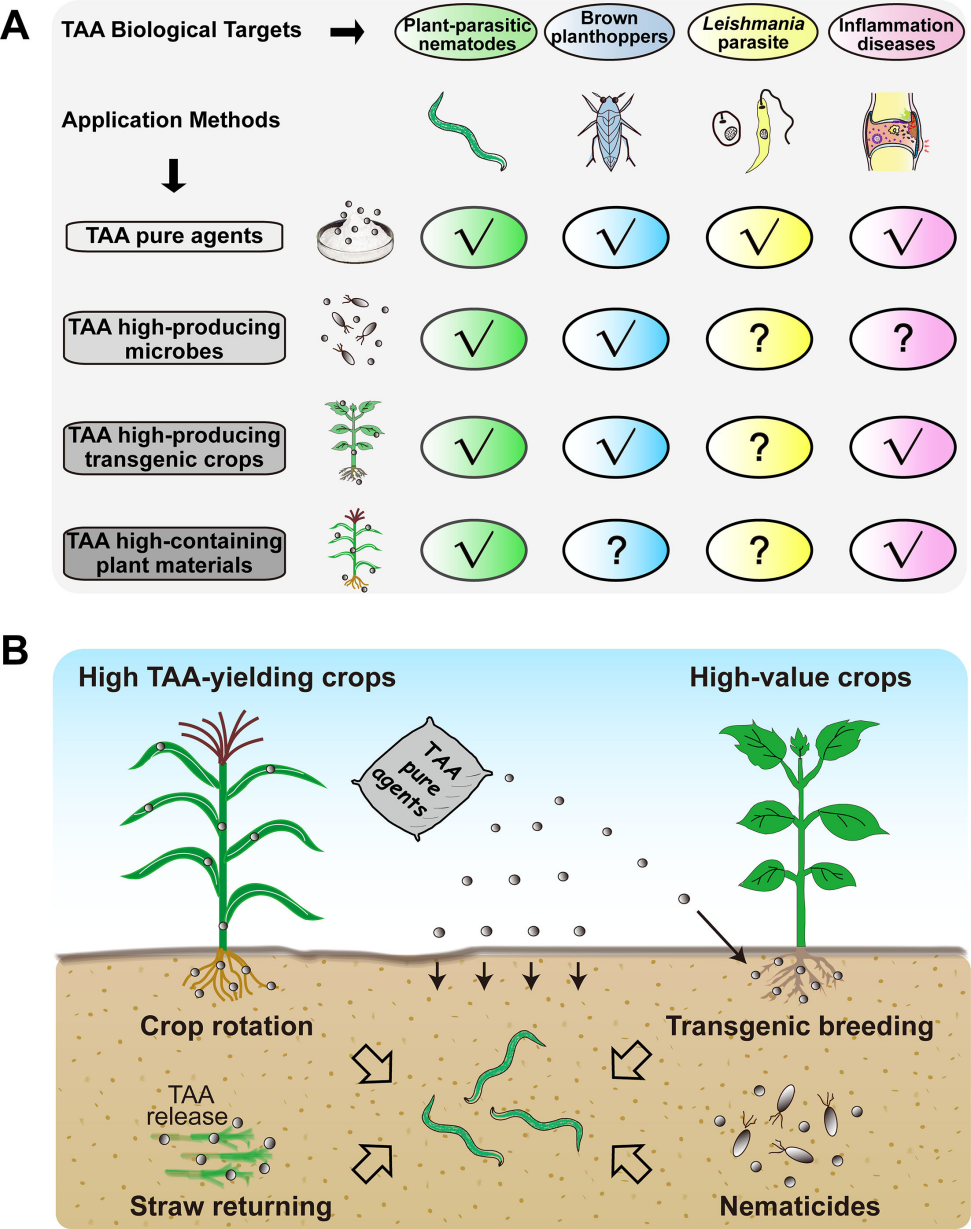
Biological targets and potential applications of TAA in agriculture and medicine. (**A**) Reported biological targets and applications for TAA. "?" symbol indicates applications that have not yet been explored or are under investigation. (**B**) Schematic diagram of a multi-pronged management strategy for TAA against plant-parasitic nematodes.

These innovations fill a key gap in our understanding of the taxonomic diversity of TAA-producing microorganisms. The newly discovered phylogenetic lineages (Fig. 6), combined with future isolates accessible by this method, construct a genetic library for AI enzymes and TAA export proteins. In the aforementioned *A. terreus* system, heterologous expression of the *tbrA* gene redirects metabolic flux toward TAA biosynthesis and is a key determinant of achieving a high titer of 60 g·L⁻¹ (21). Notably, the catalytic efficiency of TbrA is low (*k*_cat_/*K*_m_ = 0.65 s^−1^·mM^−1^) (31), which is 1,230-fold lower than the reported catalytic efficiency of AI from the *Pseudomonas* sp. WU-0701 (*k*_cat_/*K*_m_ = 800 s^−1^·mM^−1^) (29). Replacing with more efficient AI homologs has the potential to significantly increase TAA yield. Consequently, continued in-depth research on these taxa will lead to the development of higher-performance AI enzymes through subsequent natural sequence isolation or rational protein design. These variants will lay the foundation for advanced microbial systems for large-scale commercial production of TAA and promote its applications in more challenging fields beyond agricultural biocontrol and treatment (Fig. 7).

We must acknowledge the potential limitations of our screening approach. This strategy relies on TAA assimilation as a selection criterion, which may fail to identify bacteria capable of producing TAA but unable to assimilate it. This limitation does not stem from functionally distinct AIs possessing specific assimilation or synthetic activities. Rather, our previous experimental evidence confirms that all bacterial AIs identified to date share the same *in vitro* enzymatic properties and exhibit functional complementarity *in vivo* (1). Critically, TAA transporters are the key determinants of TAA-associated physiological functions. Specifically, in the biosynthetic pathway, TAA exporters are specifically designed to eliminate the competitive inhibition of aconitase in the TCA cycle by intracellular accumulation of TAA (32, 33), thereby enabling the extracellular accumulation of TAA. Conversely, during assimilation, specific importers are indispensable to deliver environmental TAA into the cell for AI-mediated carbon incorporation into central metabolism. For instance, in *B. thuringiensis* CT-43, the native expression of the TbrA enzyme and the TbrB exporter drives TAA biosynthesis, whereas the deletion of the *tbrB* gene eliminates this ability (17). Furthermore, CT-43 lacks TAA importers, thus preventing it from using TAA as the sole carbon source in minimal ACO media. In *B. velezensis* FZB42, the AI enzyme TarA, which can functionally substitute for TbrA in *Bacillus* TAA production, partners with the importer TarB, enabling the cell to specifically dedicate to TAA assimilation (1). Consequently, our method may underestimate the diversity of TAA-producing bacteria. Nevertheless, this strategy has a higher success rate and is more efficient compared to conventional methods that require individual fermentation and analysis of all environmental colonies. Future optimization of the method could be achieved by developing direct biosensor systems that respond to TAA. Such a system can integrate TAA-sensitive indicators, such as the TarR transcriptional regulator identified in *B. velezensis* FZB42 (1), with a regulon promoter to create a reporter strain that can signal the presence of TAA in the environment.

Our findings suggest that the simultaneous biosynthesis and assimilation of TAA within the same bacterial strain are quite common. Furthermore, although both pathways require AI catalysis, their activities are not intrinsically linked. This discovery is crucial because it raises several intriguing questions for future research: (i) what ecological or biological advantages does TAA production bring to these newly discovered bacteria? (ii) what molecular or environmental signals trigger the natural switches between TAA biosynthesis (e.g., for pathogenicity or self-defense) and assimilation (e.g., for nutrition adaptation)? (iii) what is the mechanism that regulates this functional balance? (iv) are these dual functions achieved by a single bifunctional AI system with differential gene expression, or do some bacteria use independent dedicated AI systems? The answers to these questions will promote microbial engineering to enhance colonization stability and biocontrol activity by regulating flexible TAA metabolic transformation.

## MATERIALS AND METHODS

### Environmental sample collection

A total of 135 environmental samples were collected from 10 provinces, including Gansu, Qinghai, Sichuan, Yunnan, Guangdong, Hubei, Shanxi, Shandong, Heilongjiang, and Liaoning, and four autonomous regions, including Xinjiang, Xizang, Ningxia, and Inner Mongolia in China. The samples are diverse, including soil, plant tissues, seawater, river water, lake water, sludge, animal feces, and sand, to ensure a broad representativeness of microbial diversity. Detailed information for each sample is provided in Table S1.

### Bacterial culture conditions

Pretreatment of environmental samples was carried out using enrichment medium of lysogeny broth (LB) medium (10 g·L⁻¹ tryptone, 10 g·L⁻¹ NaCl, 5 g·L⁻¹ yeast extract, pH 7.0).

To screen TAA-assimilating bacteria, minimal ACO solid medium was used. This medium contained 7.5 g·L⁻¹ TAA as the sole carbon source and also contained 2 g·L⁻¹ (NH_4_)_2_SO_4_, 1 g·L⁻¹ K_2_HPO_4_, 0.5 g·L⁻¹ MgSO_4_, 0.1 g·L⁻¹ FeCl_3_·6H_2_O, and 1.6% (wt·vol⁻¹) agar, with the pH adjusted to 7.0. Chemical standard TAA (purity >98%) was purchased from Tokyo Chemical Industry Co., Ltd. (Nihonbashi-Honcho, Tokyo, Japan).

In the TAA production assay, minimal CIT liquid medium containing 7.5 g·L⁻¹ citric acid as the sole carbon source, 2 g·L⁻¹ (NH_4_)_2_SO_4_, 1 g·L⁻¹ K_2_HPO_4_, 0.5 g·L⁻¹ MgSO_4_, and 0.1 g·L⁻¹ FeCl_3_·6H_2_O (pH 7.0) was used.

### Two-step screening of TAA-producing environmental bacteria

#### Step 1: high-throughput screening of TAA-assimilating bacterial isolates

One gram (solid) or 1 mL (liquid) of environmental sample was inoculated into 20 mL of LB and incubated at 37°C with vigorous shaking. After 3 h of enrichment, 1 mL of the culture supernatant was centrifuged at 10,000 × *g* for 5 min, the pellet was washed and resuspended in phosphate-buffered saline (Na_2_HPO_4_ 8 mM, NaCl 136 mM, KH_2_PO_4_ 2 mM, KCl 2.6 mM, pH 7.4), and a 400 μL aliquot was spread onto ACO plates and incubated at 37°C for 24 h. Distinct colonies were picked and inoculated into LB, and the culture density was adjusted to an OD_600_ of 0.01. Subsequently, 1 μL of each culture was streaked onto fresh ACO plates to confirm its TAA assimilation capacity.

#### Step 2: screening for TAA-producing bacterial strains

The TAA-assimilating isolates were individually inoculated into 5 mL of LB and cultured at 37°C with shaking at 200 rpm for 12 h. Subsequently, 1 mL of each culture was transferred to 100 mL of the CIT liquid medium and incubated at 37°C, 200 rpm, for 24 h. One milliliter of the culture supernatant was harvested by centrifugation, and TAA was extracted using a multi-step precipitation method with acetone and acetonitrile, as described previously (17). The final precipitate was dissolved in 0.1 mL of mobile phase (100% H_2_O with 0.1% vol·vol⁻¹ formic acid and 3 mM ammonium acetate) for TAA analysis. The strains were preserved in 25% glycerol at −80°C.

### Detection and quantification of TAA

High-resolution LC-Q-TOF-MS system (Agilent 1260 LC coupled to an Agilent G6540A Q-TOF-MS) equipped with an Agilent ZORBAX SB-Aq column (2.1 × 150 mm, 3.5 μm) was used for the detection of TAA. Analytical parameters were diode array detection at 260 nm, negative ion mode, fragmentor voltage 80 V, skimmer voltage 65 V, and a mass range of *m*/*z* 50 to 1,500. The instrument was calibrated using standard references with masses of 112.9855 and 1,033.9881 Da. The extracted ion chromatogram of TAA was analyzed using Agilent MassHunter software. The characteristic ions for TAA are the [M-H]⁻ at *m*/*z* 173.0105 and its fragment ions with *m*/*z* 129.0204 and 85.0307, respectively, corresponding to the removal of one and two carboxyl groups from the [M-H]⁻ ion. The concentration of TAA was quantitatively analyzed using HPLC with the external standard curve method.

### Species identification of TAA-producing isolates

For precise species-level identification, we carried out genome-based average nucleotide identity (ANI) analysis. Whole-genome sequencing was conducted by Bioyi Biotechnology Co., Ltd (Wuhan, Hubei, China). The obtained genome sequences of the isolates were aligned with the reference genomes of the type strains using FastANI (v1.33) with default parameters (k-mer size = 16). The results were verified using the OrthoANIu algorithm (EzBioCloud). A 95% ANI threshold was set: isolates with ANI ≥95% were assigned to the corresponding species, while those below this threshold were considered potential new species or identified only at the genus level.

### Phylogenetic tree construction based on single-copy orthologous genes

A phylogenetic tree was constructed based on single-copy orthologous genes identified using OrthoFinder (v2.5.4b) (34). The analysis included the protein sequence files (FASTA format) of 19 sequenced strains and the reference strain *B. thuringiensis* CT-43.

### Statistical analysis

The correlation between TAA assimilation capacity and TAA yield was analyzed using bivariate correlation function in IBM SPSS Statistics (v29). The Pearson correlation coefficients were calculated to determine the statistical significance of the relationship.

## Data Availability

The genome data for 19 isolated TAA-producing bacteria were deposited in the NCBI BioProject database under the accession number PRJNA1405453.

## SUPPLEMENTAL MATERIAL

Supplemental figures aem.01831-25-s0001.docx Fig. S1 to S3.

Table S1 aem.01831-25-s0002.xlsx Details of the environmental samples collected for screening TAA-producing bacteria.

Table S2 aem.01831-25-s0003.xlsx Summary of the 19 TAA-producing bacterial isolates, including their TAA yields and taxonomic identification.

ASM does not own the copyrights to Supplemental Material that may be linked to, or accessed through, an article. The authors have granted ASM a non-exclusive, world-wide license to publish the Supplemental Material files. Please contact the corresponding author directly for reuse.
