# Supplementary material for: A two-step screening platform based on trans-aconitic acid assimilation unlocks novel bacterial resources for trans-aconitic acid production
Source: Appl Environ Microbiol. 2026 Apr 13;92(5):e01831-25. doi: 10.1128/aem.01831-25 (PMC13188880; doi:10.1128/aem.01831-25)
Supplement: Supplemental figures — Fig. S1 to S3. [file aem.01831-25-s0001.docx]

***Supplementary Material for***

**A Two-Step Screening Platform Based on *Trans*-Aconitic Acid Assimilation Unlocks Novel Bacterial Resources for *Trans*-Aconitic Acid Production**

Cao Zheng^1^, Jingyi Hua^1^, Chengru Yang^1^, Bowen Sun^1^, Jingru Wu^1^, Anming Li^1^, Yujun Dai^1^, Cuiying Du^1^

^1^Hubei Province Research Center of Engineering Technology for Utilization of Botanical Functional Ingredients & Hubei Key Laboratory of Resource Utilization and Quality Control of Characteristic Crops, College of Life Science and Technology, Hubei Engineering University, Xiaogan, Hubei, China.

Corresponding authors: Cuiying Du, College of Life Science and Technology, Hubei Engineering University, No.272 Jiaotong Avenue, Xiaogan 432000, Hubei, China. Email: ducuiying.123@163.com.

| **Page** | **Content** |
| --- | --- |
| **3** | **Fig. S1** Molecular basis of the dual bacterial physiological mechanisms of TAA metabolism |
| **4** | **Fig. S2** Mass spectra of TAA extracts from the culture supernatants of 18 TAA-producing isolates |
| **5** | **Fig. S3** HPLC chromatograms of TAA production in the culture supernatant of 19 TAA-producing bacterial strains |
| **6** | **Titles of Supplementary Tables for separately uploaded excel files** |


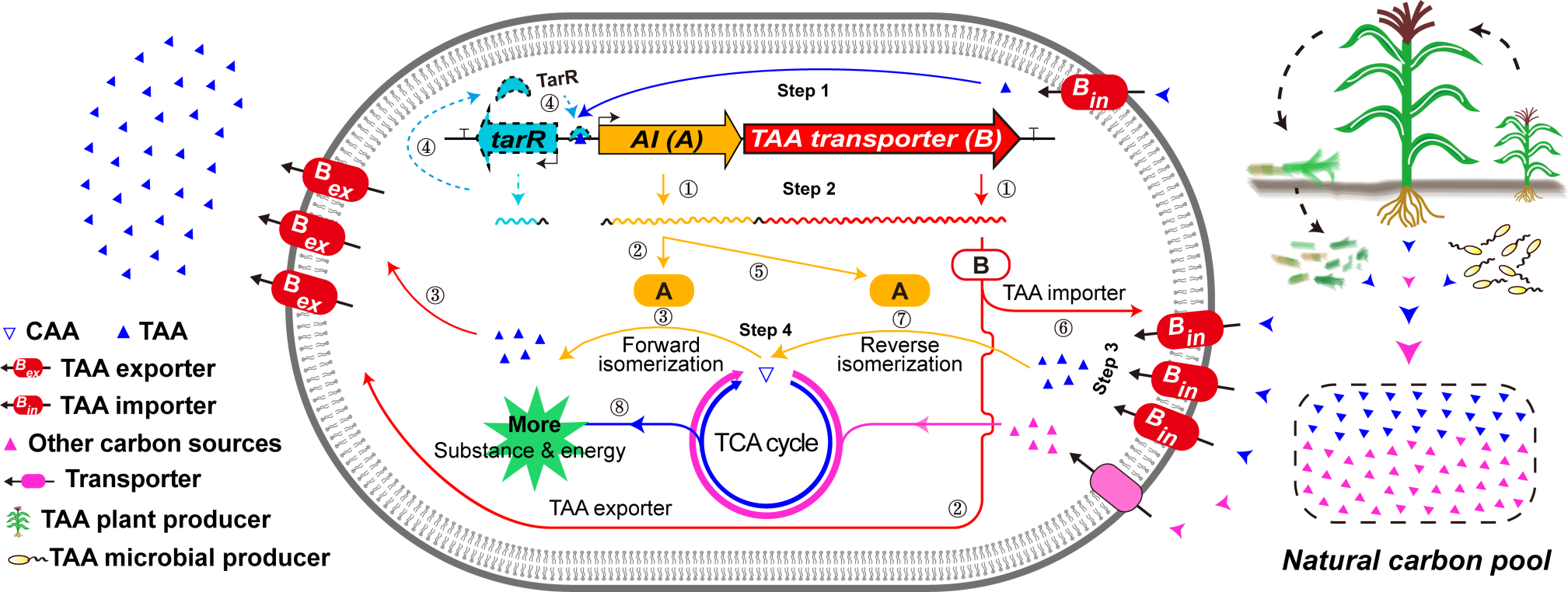


**Fig. S1** **Molecular basis of the dual bacterial physiological mechanisms of TAA metabolism.** In *B. thuringiensis* CT-43, constitutive expression of the operon encoding AI (*orange*) and the TAA extracellular transporter (*B_ex_*, *red*) (arrows ① and ②) drives the sustained production of intracellular TAA and its extracellular accumulation (arrow ③) (Reference 17 in the main text). In contrast, in *B. velezensis* FZB42, the assimilation operon encoding AI (*orange*) and the TAA intracellular transporter (*B_in_*, *red*) exhibits an inducible expression pattern: Step 1, Sensing. TAA from the environment enters the cytoplasm via low levels of *B_in_*. Step 2. Activation. The cytoplasmic transcriptional activator TarR senses and binds to TAA (arrow ④), triggering strong expression of the assimilatory operon (arrows ①, ⑤, and ⑥). Step 3. Transport. *B_in_* efficiently imports TAA from the environment. Step 4. Isomerization. AI converts TAA to CAA (arrow ⑦), and imports carbon into the TCA cycle to support more biosynthesis and energy production (arrow ⑧) (Reference 1 in the main text).

**
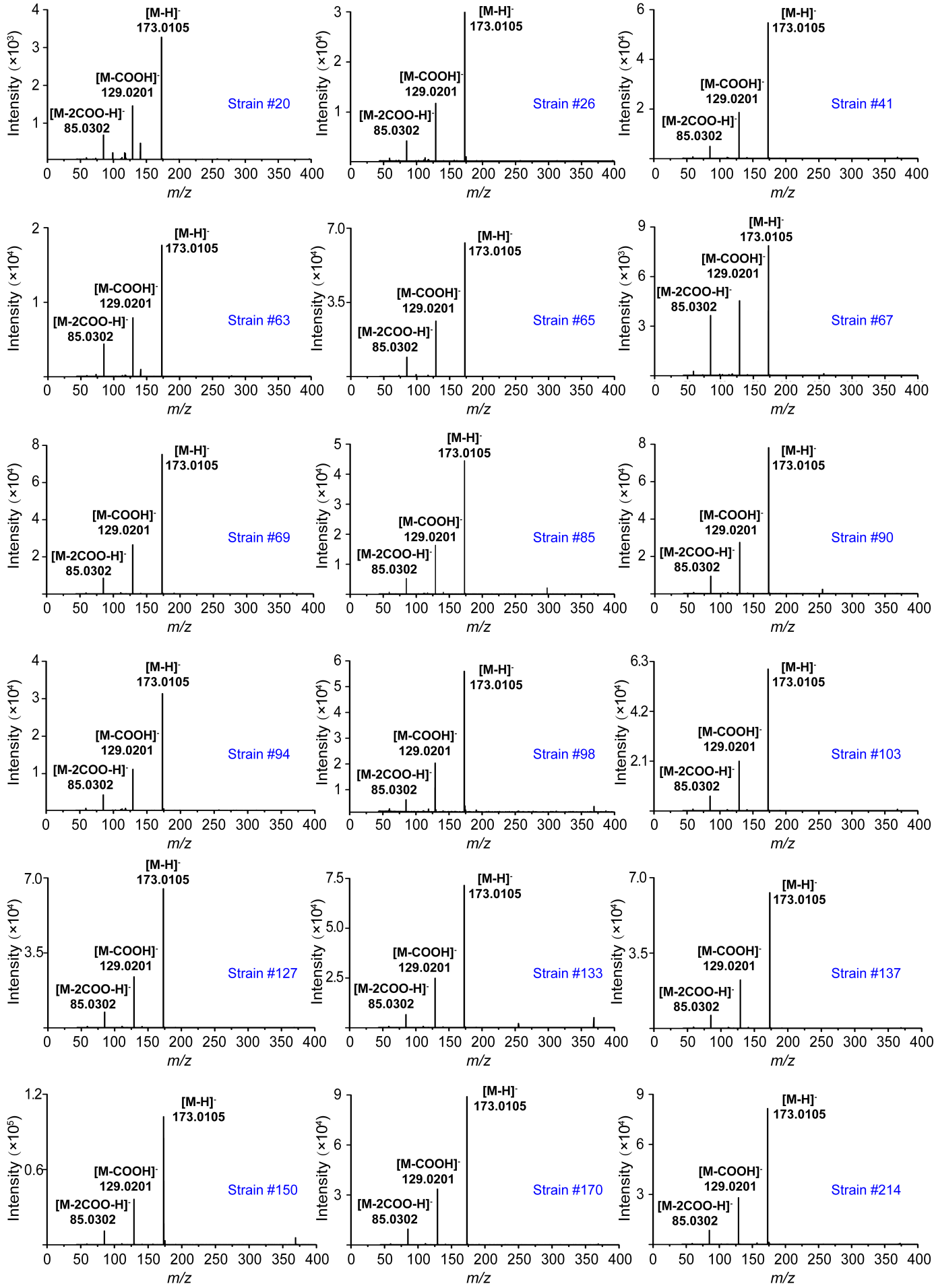
Fig. S2 Mass spectra of TAA extracts from the culture supernatants of 18 TAA-producing isolates. Analysis of the extraction ion chromatograms (EICs) revealed the presence of [M-H]⁻ ions and their characteristic decarboxylation products [M-COOH]⁻ at ‘*m/z*’ 129.0201, and [M-2COO-H]⁻ at ‘*m/z*’ 85.0302.**

**
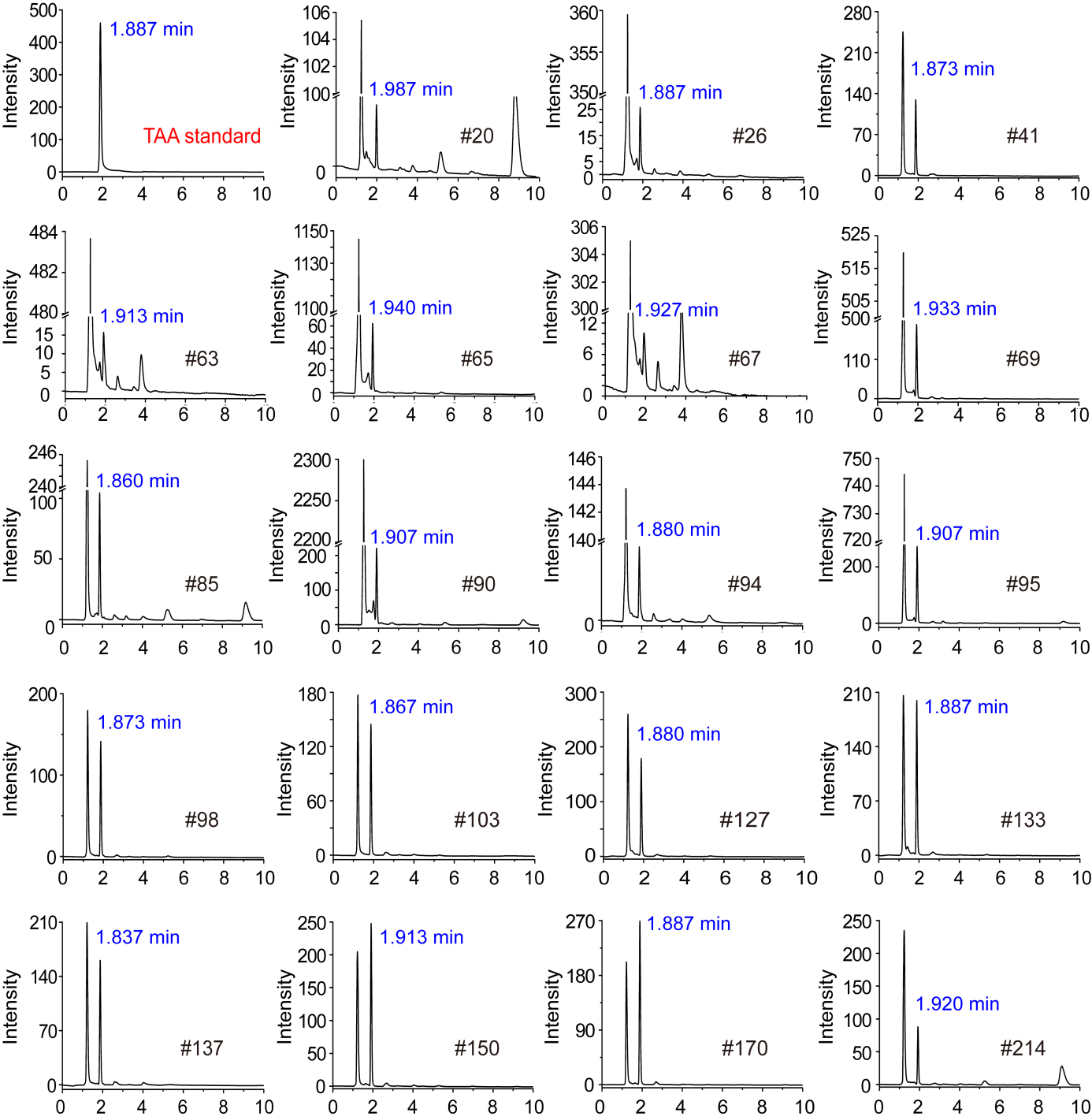
**

**Fig. S3** **HPLC chromatograms of TAA production in the culture supernatant of 19 TAA-producing bacterial strains**

**Titles of Supplementary Tables (separately uploaded excel files)：**

**Table S1** Details of the environmental samples used for screening TAA-producing bacteria.

**Table S2** Summary of 19 TAA-producing bacterial isolates, including their TAA yields and classification

**The superscript numbers** in columns for order, family, and genus correspond to the number of novel taxa of TAA-producing bacteria identified in this study.

Species identification was performed by aligning sequencing reads to a reference genome in the Genome Taxonomy Database (GTDB) using Sylph (v 0.6.1).

The table columns are defined as follows:

**Species**, the taxonomic name of the best-matching reference genome;

**Relative Abundance**, the proportion of reads assigned to this species;

**Sequence Abundance**, the number of reads aligned to this species;

**ANI**, the average nucleotide identity between the query genome and the reference genome;

**Coverage**, the breadth of alignment coverage of the reference genome;

**Genome_id**, the unique identifier of the closest matching reference genome in GTDB.

*Note: Isolate #85 was identified as a potential new species. This designation was based on ANI analysis because its ANI value against all known reference genomes was below the species threshold (<95%).*
